# Integrative analysis of genome-wide association studies and gene expression analysis identifies pathways associated with rheumatoid arthritis

**DOI:** 10.18632/oncotarget.7390

**Published:** 2016-02-14

**Authors:** Mingming Zhang, Hongbo Mu, Hongchao Lv, Lian Duan, Zhenwei Shang, Jin Li, Yongshuai Jiang, Ruijie Zhang

**Affiliations:** ^1^ College of Bioinformatics Science and Technology, Harbin Medical University, Harbin, China; ^2^ College of Science, Northeast Forestry University, Harbin, China

**Keywords:** rheumatoid arthritis, pathway analysis, single nucleotide polymorphism, gene expression, Pathology Section

## Abstract

Rheumatoid arthritis (RA) is a complex and systematic autoimmune disease, which is usually influenced by both genetic and environmental factors. Pathway analyses based on a single data type such as microarray data or SNP data have successfully revealed some biology pathways associated with RA. However, we found that the pathway analysis based on a single data type only provide limited understanding about the pathogenesis of RA. Gene-disease association is usually caused by many ways, such as genotype, gene expression and so on. Therefore, the integrative analysis method combining multiple levels of evidence can more precisely and comprehensively identify the pathway associations. In this study, we performed a pathway analysis by integrating GWAS and gene expression analysis to detect the RA-related pathways. The integrative analysis identified 28 pathways associated with RA. Among these pathways, 18 pathways were also found by both GWAS and gene expression analysis, 7 pathways are novel RA-related pathways, such as B cell receptor signaling pathway, Toll-like receptor signaling pathway, Fc gamma R-mediated phagocytosis and so on. Compared with pathway analyses using only one type genomic data, we found integrative analysis can increase the power to identify the real associations and provided more stable and accurate results. We believe these results will contribute to perform future genetic studies in RA pathogenesis and may promote the development of new therapeutic strategies by targeting these pathways.

## INTRODUCTION

Rheumatoid arthritis (RA) is a common, chronic autoimmune disease, which characterized by nonspecific inflammation of the peripheral joints. It affects 1% of the world's population, and it occurs more frequently in women than in men [1, 2]. In later disease stages, affected joint and surrounding tissues exhibit progressive destruction, and cause dysfunction of joint and disable. It is generally accepted that RA is associated with both genetic and environmental factors, but the etiology of RA is still not very clear to date.

Genetic factors are known to affect disease susceptibility. In recent years, there are many researches to identify the genetic factors (loci, genes and pathways) related to RA. Genome-wide association studies (GWAS) is one of the main methods in finding genetic variants. Many GWAS have been successfully applied to RA and have identified large number of single nucleotide polymorphisms (SNPs) related to RA [3-8]. But these loci account for a small proportion of genetic variants and can not dissect and explain the complex molecular mechanisms of RA. Besides, some SNPs which do not reach the significant threshold but have modest effects on RA were not identified by GWAS.

Besides genetic association studies, there are still a lot of bioinformatics analysis methods to help us to understand the molecular mechanism of RA. Genome-wide gene expression analysis as a tool to investigate pathogenesis of RA has found many candidate genes for RA. In 2004, Devauchelie et al. identified differential expression genes between RA and osteoarthritis (OA) by DNA microarray analysis [9]. In 2006, Haas et al. found 1,163 significantly differentially expressed genes in RA twin compared with healthy twin [10]. Kim et al. performed a gene expression analysis and found G0S2 plays as a biomarker to predict response to anti-TNF therapy in patients with RA [11]. Xu et al. found P53 may be involved in the progression of RA and osteoarthritis via targeting downstream EGFR and DUSP1 respectively based on gene expression profiles [12]. Several other publications also identified some RA-specific gene signatures by gene expression analysis [13, 14] [15]. These studies show that microarray analysis is a useful tool to detect RA-related genes and could help in identifying novel therapeutic targets. However, some genes that do not change at the level of gene expression but are really associated with RA are still not found.

It is well known that genes do not work in isolation, instead, complex biology pathways and molecular networks are usually involved in disease susceptibility and disease progression [16]. So gene set or pathway association analyses which identify variation in the pathway or biology function related to the disease have been developed. Pathway association analysis can reduce the false positives and uncertainty around causal genes by analyzing associations between pathways and the disease. In addition, the pathways detected by pathway analysis can also help us explain underlying molecular mechanism of the disease.

There are many pathway analyses studies of RA based on a single data type, for example, SNP data or gene expression data, which have successfully revealed some biology pathways associated with RA [17] [2, 18-20]. However, pathway analysis of RA which integrates GWAS and gene expression information is still lacking. Some valuable associations may be ignored by a single data type analyses. For example, some genes with only genetic variations can't be identified in gene expression studies. Similarly, genes with only expression changes also are discarded in GWAS. It is easily found that the pathway analysis based on a single data type only provide limited understanding about the development of RA. Therefore, integrating GWAS and gene expression information into pathway association analysis can provided more accurate and stable evidence to explain pathogenesis of RA.

In this study, we integrated SNP dataset and gene expression information to identify the pathways related to RA. This multi-leave procedure consists of six computation steps: (1) compute the *P*-value of a SNP by trend test and assess gene-wise association value based on SNP dataset; (2) compute the gene expression *P*-value of every gene based on gene expression data; (3) compute the gene association score by integrating GWAS and gene expression; (4) compute the pathway association score based on SNP dataset, gene expression data, and integrated datasets respectively; (5) compute the significance of association between the pathways and RA by permutation test; (6) identify the pathways related to RA after multiple testing corrections. By comparing the results, we found integrative analysis can provide more stable results and also revealed novel pathway associations.

## RESULTS

### The pathway analysis results based on GWAS

In the WTCCC SNP dataset, there were 370,227 SNPs passed quality control. After mapping SNPs to genes, we found that there were 32,591 genes that contained at least one SNP. We used 1000 genome dataset from Europe to correct the LD and obtained tagSNPs of all 32,591 genes. The smallest *P-*value of all tagSNPs in a gene was chosen to be the gene-wise association value.

The 234 pathway risk scores were computed by the Fisher's method. The RA-related pathways were identified by the permutation test and FDR correction. The 19 pathways whose *q*-values were less than 0.05 after FDR correction were identified as RA-related pathways based on GWAS. The detailed information of these pathways is presented in Table 1.

**Table 1:** The RA-related pathways based on SNP dataset (FDR<0.05)

Pathway ID	Pathway name	*P*-value	FDR	Function class
hsa04145	*Phagosome	0	0	Cellular Processes; Transport and catabolism
hsa04514	*Cell adhesion molecules (CAMs)	0	0	Environmental Information Processing; Signaling molecules and interaction
hsa04612	*Antigen processing and presentation	0	0	Organismal Systems; Immune system
hsa04672	*Intestinal immune network for IgA production	0	0	Organismal Systems; Immune system
hsa04940	*Type I diabetes mellitus	0	0	Human Diseases; Endocrine and metabolic diseases
hsa05140	*Leishmaniasis	0	0	Human Diseases; Infectious diseases
hsa05145	*Toxoplasmosis	0	0	Human Diseases; Infectious diseases
hsa05150	*Staphylococcus aureus infection	0	0	Human Diseases; Infectious diseases
hsa05168	*Herpes simplex infection	0	0	Human Diseases; Infectious diseases
hsa05310	*Asthma	0	0	Human Diseases; Immune diseases
hsa05320	*Autoimmune thyroid disease	0	0	Human Diseases; Immune diseases
hsa05330	*Allograft rejection	0	0	Human Diseases; Immune diseases
hsa05332	*Graft-versus-host disease	0	0	Human Diseases; Immune diseases
hsa05416	*Viral myocarditis	0	0	Human Diseases; Cardiovascular diseases
hsa05164	*Influenza A	1.00E-05	0.00013	Human Diseases; Infectious diseases
hsa05162	*Measles	0.00015	0.001831	Human Diseases; Infectious diseases
hsa05323	*Rheumatoid arthritis	0.00038	0.004365	Human Diseases; Immune diseases
hsa05152	*Tuberculosis	0.00102	0.011064	Human Diseases; Infectious diseases
hsa05322	*Systemic lupus erythematosus	0.00468	0.048095	Human Diseases; Immune diseases

* Pathways were reported as RA-related pathways in previous literatures.

According to the KEGG classifications, we found these pathways were mainly involved in immune system (*n*=8), infectious diseases (*n*=7), signaling molecules and interaction (*n*=1), transport and catabolism (*n*=1), endocrine and metabolic diseases (*n*=1) and cardiovascular diseases (*n*=1). All of these pathways are reported as RA-related pathway in the previous studies. It is indicated our results are consistent with previously published studies [17, 20-22].

### The pathway analysis results based on gene expression analysis

The gene expression data of 53 individuals (33 RA and 20 controls) were normalized by “affy” package in R software. Then the probes were mapped to the genes according to platform information. After data preprocessing, 12,752 genes were for following analysis. The differential expression *P*-values of the 12,752 genes were computed by *t*-test. Then, we computed the 234 pathway risk scores using the Fisher's method and identified the RA-related pathways by the permutation test and FDR correction. Finally, the 33 pathways whose *q*-values were less than 0.05 after FDR correction were identified as RA-related pathways based on gene expression data. The detailed information of these pathways is presented in Table 2.

**Table 2:** The RA-related pathways based on gene expression dataset (FDR<0.05)

Pathway ID	Pathway name	*P*-value	FDR	Function class
hsa04062	*Chemokine signaling pathway	0	0	Organismal Systems; Immune system
hsa04145	*Phagosome	0	0	Cellular Processes; Transport and catabolism
hsa04514	*Cell adhesion molecules (CAMs)	0	0	Environmental Information Processing; Signaling molecules and interaction
hsa04612	*Antigen processing and presentation	0	0	Organismal Systems; Immune system
hsa04640	*Hematopoietic cell lineage	0	0	Organismal Systems; Immune system
hsa04650	*Natural killer cell mediated cytotoxicity	0	0	Organismal Systems; Immune system
hsa04672	*Intestinal immune network for IgA production	0	0	Organismal Systems; Immune system
hsa04940	*Type I diabetes mellitus	0	0	Human Diseases; Endocrine and metabolic diseases
hsa05140	*Leishmaniasis	0	0	Human Diseases; Infectious diseases
hsa05150	*Staphylococcus aureus infection	0	0	Human Diseases; Infectious diseases
hsa05152	*Tuberculosis	0	0	Human Diseases; Infectious diseases
hsa05162	*Measles	0	0	Human Diseases; Infectious diseases
hsa05168	*Herpes simplex infection	0	0	Human Diseases; Infectious diseases
hsa05320	*Autoimmune thyroid disease	0	0	Human Diseases; Immune diseases
hsa05323	*Rheumatoid arthritis	0	0	Human Diseases; Immune diseases
hsa05330	*Allograft rejection	0	0	Human Diseases; Immune diseases
hsa05332	*Graft-versus-host disease	0	0	Human Diseases; Immune diseases
hsa05340	Primary immunodeficiency	0	0	Human Diseases; Immune diseases
hsa05416	*Viral myocarditis	0	0	Human Diseases; Cardiovascular diseases
hsa04660	*T cell receptor signaling pathway	1.00E-05	9.95E-05	Organismal Systems; Immune system
hsa05310	*Asthma	2.00E-05	0.000181	Human Diseases; Immune diseases
hsa05164	*Influenza A	2.00E-05	0.000189	Human Diseases; Infectious diseases
hsa04662	B cell receptor signaling pathway	4.00E-05	0.000346	Organismal Systems; Immune system
hsa04666	Fc gamma R-mediated phagocytosis	5.00E-05	0.000414	Organismal Systems; Immune system
hsa05145	*Toxoplasmosis	9.00E-05	0.000716	Human Diseases; Infectious diseases
hsa04620	Toll-like receptor signaling pathway	0.00011	0.000842	Organismal Systems; Immune system
hsa04380	Osteoclast differentiation	0.00014	0.001031	Organismal Systems; Development
hsa04142	Lysosome	0.00244	0.017334	Cellular Processes; Transport and catabolism
hsa03030	*DNA replication	0.00326	0.021616	Genetic Information Processing; Replication and repair
hsa05110	Vibrio cholerae infection	0.00317	0.021744	Human Diseases; Infectious diseases
hsa04670	*Leukocyte transendothelial migration	0.00431	0.027656	Organismal Systems; Immune system
hsa04110	*Cell cycle	0.00521	0.032386	Cellular Processes; Cell growth and death
hsa04664	Fc epsilon RI signaling pathway	0.00778	0.046896	Organismal Systems; Immune system

* Pathways were reported as RA-related pathways in previous literatures. Italic pathways were also detected based on GWAS.

We found the majority of these 33 pathways are related to immune system (*n*=17), infectious diseases (*n*=8), cellular processes (*n*=3). Among these pathways, there are 24 pathways to be found respectively as RA-related pathways in the pervious published studies [2, 21, 23, 24]. There are 18 pathways to be identified by SNP dataset and 10 novel pathways which were not reported in pervious literatures.

### The pathway analysis results based on integration GWAS and gene expression data

We chose the 11,922 genes which have both gene-wise association value of GWAS and the differential expression value. We combined gene-wise association value of GWAS and the differential expression value to generate a single gene association score by Fisher's combined method. Then we translated the gene association score into the of chi-square statistic *P*-value and defined this *P*-value as the single gene association *P*-value after combination GWAS and gene expression data. Then, we computed the 234 KEGG pathway risk scores using the Fisher's method and identified the RA-related pathways by the permutation test and FDR correction.

Finally, the 28 pathways (FDR< 0.05) after FDR correction were identified as RA-related pathways based on integration GWAS and gene expression data. Among these pathways, they were mainly involved in immune system (*n*=16), infectious diseases (*n*=7), signaling molecules and interaction (*n*=1), transport and catabolism (*n*=1), endocrine and metabolic diseases (*n*=1), development (*n*=1) and cardiovascular diseases (*n*=1). The detailed information of these pathways is presented in Table 3. There are 18 same pathways to be found by pathway analysis of GWAS and gene expression data respectively. Integrative analysis also predicted 7 novel RA-related pathways which are natural killer cell mediated cytotoxicity, primary immunodeficiency, Hematopoietic cell lineage, Fc gamma R-mediated phagocytosis, B cell receptor signaling pathway, osteoclast differentiation and Toll-like receptor signaling pathway. According to function classification of KEGG, the other six pathways belong to immune system except osteoclast differentiation pathway.

**Table 3:** The RA-related pathways identified by integrating GWAS and gene expression (FDR<0.05)

Pathway ID	Pathway name	Combined_*P*-value	FDR	Function class
hsa04062	*Chemokine signaling pathway	0	0	Organismal Systems; Immune system
hsa04145	*Phagosome	0	0	Cellular Processes; Transport and catabolism
hsa04514	*Cell adhesion molecules (CAMs)	0	0	Environmental Information Processing; Signaling molecules and interaction
hsa04612	*Antigen processing and presentation	0	0	Organismal Systems; Immune system
hsa04650	Natural killer cell mediated cytotoxicity	0	0	Organismal Systems; Immune system
hsa04672	*Intestinal immune network for IgA production	0	0	Organismal Systems; Immune system
hsa04940	*Type I diabetes mellitus	0	0	Human Diseases; Endocrine and metabolic diseases
hsa05140	*Leishmaniasis	0	0	Human Diseases; Infectious diseases
hsa05145	*Toxoplasmosis	0	0	Human Diseases; Infectious diseases
hsa05150	*Staphylococcus aureus infection	0	0	Human Diseases; Infectious diseases
hsa05152	*Tuberculosis	0	0	Human Diseases; Infectious diseases
hsa05162	*Measles	0	0	Human Diseases; Infectious diseases
hsa05164	*Influenza A	0	0	Human Diseases; Infectious diseases
hsa05168	*Herpes simplex infection	0	0	Human Diseases; Infectious diseases
hsa05310	*Asthma	0	0	Human Diseases; Immune diseases
hsa05320	*Autoimmune thyroid disease	0	0	Human Diseases; Immune diseases
hsa05323	*Rheumatoid arthritis	0	0	Human Diseases; Immune diseases
hsa05330	*Allograft rejection	0	0	Human Diseases; Immune diseases
hsa05332	*Graft-versus-host disease	0	0	Human Diseases; Immune diseases
hsa05340	Primary immunodeficiency	0	0	Human Diseases; Immune diseases
hsa05416	*Viral myocarditis	0	0	Human Diseases; Cardiovascular diseases
hsa04660	*T cell receptor signaling pathway	2.00E-05	0.000175	Organismal Systems; Immune system
hsa04640	Hematopoietic cell lineage	3.00E-05	0.000251	Organismal Systems; Immune system
hsa04666	Fc gamma R-mediated phagocytosis	0.00029	0.002325	Organismal Systems; Immune system
hsa04662	B cell receptor signaling pathway	0.00042	0.003233	Organismal Systems; Immune system
hsa04380	Osteoclast differentiation	0.00074	0.005477	Organismal Systems; Development
hsa04620	Toll-like receptor signaling pathway	0.00111	0.007912	Organismal Systems; Immune system
hsa04670	*Leukocyte transendothelial migration	0.00651	0.044744	Organismal Systems; Immune system

* Pathways were reported as RA-related pathways in previous literatures.

We found that pathways which were identified by pathway analyses based on GWAS and gene expression analysis were not entirely consistent. This is due to that single data type analyses only give limited genetic variation information of RA and maybe ignore some valuable association. Therefore, the pathways identified by single data type analyses are not comprehensive. Our results also indicated pathway analysis of integrating GWAS and gene expression analysis can detect stable and precise pathways which will give us an insight into complex molecular mechanisms of RA.

## DISCUSSION

RA is a complex and chronic autoimmune disease which is affected by multiple genes and environmental factors. Recently, many pathway analysis based on a single data type appeared. However, the results of these studies are varied. To identify the comprehensive and stable pathways, we carried out a pathway analysis by integrating GWAS and gene expression analysis.

In the pervious genetics studies, the authors usually selected the significant SNPs or genes by the given threshold and performed pathway analysis by using SNPs or genes which reached the threhold. The pathways identified by this method may be really associated with RA. However, some SNPs or genes which did not reach the threshold but were related to RA were usually ignored. Therefore, the moderate-effect pathways which contained many modest-effect SNPs or genes can not be identified by threshold methods. In order to overcome this limitation, we didn't scan the SNPs or genes by the threshold, but used all of genes for the following analysis.

We have not only identified some pathways which were reported to be associated with RA in the previous studies, but also some hitherto unrecognized cellular pathways, including natural killer cell mediated cytotoxicity, primary immunodeficiency, hematopoietic cell lineage, Fc gamma R-mediated phagocytosis, B cell receptor signaling pathway, osteoclast differentiation and toll-like receptor signaling pathway. These novel pathways belonged to immune system according to KEGG function classification. At present, there was no evidence of association between these pathways and RA in previous studies. But it is known that RA is a typical autoimmune disease, so we infer RA may be associated with these immune pathways very likely. Therefore, it is easy to see that the integration analysis of GWAS and gene expression can provide more comprehensive results.

We identified 16 pathways which were related to immune system. In the present studies, there was no evidence to elucidate the relationship between Chemokine signaling pathway and RA until 2015. In 2015, zhang et al. used real-time polymerase chain reaction to determine gene expressions and found the chemokine signaling pathway was involved in CCL2 expression in RA patient peripheral blood and synovial tissues [25]. The same year, Lv et al. used a random walk strategy based on the subpathway- subpathway interaction network to prioritize risk subpathways and also found chemokine signaling pathway was a risk pathway to RA [26]. We also found the association between this pathway and RA by integration analysis. B cell receptor signaling pathway is an immune pathway. To date, there were no studies to indicate the relationship between B cell receptor signaling pathway and RA. But Xu et al. investigated the microRNA (miRNA) expression pattern in synovial fluid from patients with knee osteoarthritis (OA) and found B cell receptor signaling pathway was primarily unregulated [27]. Wu et al. performed a differential expression analysis of microRNA expression profiles of extracellular vesicles in nasal mucus from patients with allergic rhinitis and identified 35 differential expressed microRNAs and 32 significantly enriched KEGG pathways. Among 32 pathways, B cell receptor signaling pathway (*P* = 3.709E-09) was one of the most prominent pathways [28]. Guo et al. carried out a microRNA analysis in peripheral blood lymphocytes in experimental autoimmune uveitis (EAU) rats versus control and found B cell receptor signaling pathway was closely associated with EAU [29]. The evidence suggests that B cell receptor signaling pathway may be associated with autoimmune disease and inflammatory disorder. Further genetics researches or animal experiments are required to confirm the relation between B cell receptor signaling pathway and RA. We believe that our results will contribute to perform future genetic studies in RA pathogenesis and may promote the development of new therapeutic strategies by targeting these pathways.

We identified 7 pathways which were related to infectious disease. These seven pathways are identified respectively by GWAS, gene expression analysis and integration analysis. Among these pathways, Influenza-A pathway was not found as RA-related pathway in previously published studies until 2013. Sharma et al. performed a multistage integrative pathway analysis on RA and firstly detected a relation between the influenza-A pathway and RA (*P* = 2.0E-04) [21]. Besides, he also found Measles pathway was significantly associated with RA (*P* = 1.0E-03) [21]. Peter et al. developed a model-based approach to pathway analysis and found the relationship between RA and “Measles” pathway genes taken part in immune responses caused by measles infection [30]. In the same year, Liu et al. performed a pathway and network analysis of large-scale RA GWAS and also identified Measles pathway as the most significant pathway associated with RA (*P* = 1.57E-08) [24]. In addition, there was no evidence to indicate the links between Measles pathway and RA in the previous studies. We also identified Measles pathway as RA-related pathway by integrative analysis of GWAS and gene expression analysis. Further researches are required to uncover the mechanism of the interaction between Measles and RA.

In our research, we performed a pathway analysis by integrating GWAS and gene expression analysis and detected some pathways related to RA. We found integrative analysis did not only detect RA-associated pathways which had confirmed in previous studies, but also can identify some novel RA-related pathways. However, in order to reveal the pathogenesis of RA, we are required to integrate more types of data into pathway analysis and perform genetic studies or experiments to prove the relationship between the novel pathways and RA in further research.

## MATERIALS AND METHODS

### Data

GWAS SNP data came from WTCCC RA datasets. The dataset included 459,446 autosomal SNPs from 12,468 subjects (1,860 cases and 10,608 controls). The individuals from the dataset are Caucasian. SNPs with a minor allele frequency (MAF) <0.01 or a *P-*value <0.001 for Hardy-Weinberg equilibrium (HWE) or a genotype percent <0.25 were excluded from the analysis [20]. After quality control, WTCCC dataset included 448,033 SNPs.

Gene expression data are from three microarray datasets in Gene Expression Omnibus (GEO) database. The three microarray datasets are GSE55235, GSE55457 and GSE55584. Their platform is GPL96 (Affymetrix HG-U133A). The individuals of three datasets are Caucasian. In this study, 53 individuals are contained, including 33 RA patients and 20 healthy individuals. The detailed information of individuals is shown in Table 4.

**Table 4:** The number of the samples in three microarray datasets

Dataset	RA	Control	Total	Platform
GSE55235	10	10	20	Affymetrix HG-U133A
GSE55457	13	10	23	Affymetrix HG-U133A
GSE55584	10	0	10	Affymetrix HG-U133A
Total	33	20	53	

Gene data were downloaded at the National Center for Biotechnology Information (ftp://ftp.ncbi.nlm.nih.gov/genomes/MapView/Homo_sapiens/sequence/BUILD.37.3/). There are altogether 34,402 autosomal genes for the following analysis. The detailed information of these genes was extracted, such as ‘gene ID’, ‘chromosome ID’, ‘gene start position’, ‘gene end position’ and so on. Then we map SNPs to genes according to the information.

Pathway data were obtained from the Kyoto Encyclopedia of Genes and Genomes (KEGG) [31]. The KEGG pathway database is a common bioinformatics resource that provides path graph of molecular interactions, chemical reactions, and gene relations. It has been widely used for the systematic analysis of gene functions that involve molecular interaction in complex diseases. There are in total 253 pathways in KEGG. To avoid testing overly narrow or broad function categories, we selected 234 human KEGG pathways that contained at least 5 and at most 200 genes [32].

### Assessing gene-wise association value based on SNP dataset

We used the Cochran-Armitage trend test to calculate *P-*values of all SNPs in the WTCCC datasets. Then we mapped a SNP to a gene if it was located within the gene or 50kb immediately upstream or downstream. All the SNPs from WTCCC RA datasets were mapped to 34,402 autosomal genes and their extended gene intervals.

**Figure 1:**
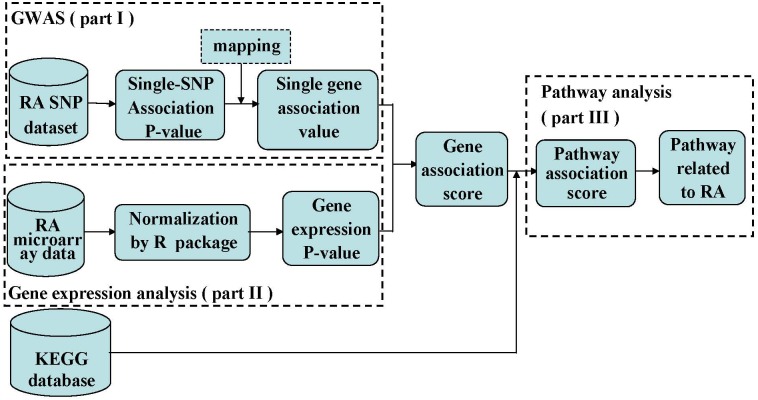
The workflow of integrative GWAS and gene expression analysis

Linkage disequilibrium is an important problem that should be considered in pathway analysis. In order to make the results more accurate, we carried out a LD correction. Because our WTCCC SNP data are not raw SNP genotype data, we chose 1000 genomes data from Europe as a reference dataset to correct LD. We obtained the tagSNPs of each gene by Haploview software when *r*^2^>0.8. We chose the smallest *P*-value of all tagSNPs in this gene to assess the gene-wise association value. See part I of workflow (Figure 1).

### Computing the differential expression value based on gene expression data

We downloaded the cel files of the three microarray datasets (GSE55235, GSE55457 and GSE55584) from GEO database. Then we mixed the three datasets together and obtained 53 individuals, including 33 RA patients and 20 controls. The data were normalized by affy package in R software. Then the probes were mapped to the genes according to platform information.

The differential expression score reflects the degree of a differentially expressed gene between cases and controls. There are many statistical methods to compute the differential expression score, such as *t*-test, analysis of variance and so on. In this study, we chose *t*-test method to analyze the microarray data after normalization and compute the differential expression *P*-values of all genes. See part II of workflow (Figure 1).

### Calculating the gene association *P*-value by integrating GWAS and gene expression analysis

We chose the genes which have both gene-wise association value based on SNP dataset and the differential expression value. We combined gene-wise association value of GWAS and the differential expression value to generate a single gene association score by Fisher's combined method. For example:

$$\begin{array}{cc} g_{i}=-2\overset{j=1}{\underset{k}{\sum }}\log(p_{j}) & k=2 \end{array}$$ (1)

Where *g_i_* is a single gene association score of *i*-th gene by integrating GWAS and gene expression. *P*_i1_, *P*_i2_ are gene-wise association value of GWAS and the differential expression value based on gene expression data.

Because *g_i_* follows *X*^2^(2K) distribution [33], we transformed the *g_i_* into the probability of chi-square distribution. The probability represented gene association *P*-value by integrating GWAS and gene expression and marked as *P_i_*.

### Calculating the pathway association score

It was known that the pathways were composed of genes. The cumulative effect of DNA variation in the pathway leads to the association of the pathway with susceptibility to disease. Thus, we calculated pathway association scores based on integrative gene association *P*-value. We still adopted Fisher's combination method to calculate pathway association scores for 234 human pathways. See part III of workflow (Figure 1).

### Permutation test

In order to further determine whether a pathway was significantly associated with RA, we performed a permutation test by shuffling the pathway labels. The null hypothesis is that there is no association between the pathway and RA. We counted the number of non-redundant genes that had association *P*-value in all 234 pathways. Then, the 234 pathway association scores were calculated by Fisher's method. Specifically, for a pathway, the pathway association score was denoted as *S_true_*. Next, we randomly chose the same number of genes as the number of genes involved in calculating this pathway association score from all non-redundant genes, calculated the random pathway association score by Fisher's method and denoted it as *S_random_*. This process was repeated 100,000 times in order to achieve sufficient randomization. The random pathway association score (*S_random_*) formed an estimated background distribution of this pathway. The smaller gene association *P*-value, the pathway association score is greater. The greater the pathway association score, the more associated the pathway is with RA. A *P_permutation_* was computed for each pathway by counting the number of permutations that have *S_random_* more than *S_true_* divided by the total number of permutations.

### Multiple testing corrections

Multiple hypothesis tests had been used to identify pathways related to RA, but they could increase type I errors and the false-positive error rate. Therefore, we adopted multiple testing corrections to solve this problem. A threshold 0.05 of *q*-value (FDR) was used to identify RA-realted pathways.

The workflow of this study was shown in Fig. 1.
